# Exogenous Glutathione Enhances Mercury Tolerance by Inhibiting Mercury Entry into Plant Cells

**DOI:** 10.3389/fpls.2017.00683

**Published:** 2017-05-01

**Authors:** Yeon-Ok Kim, Hyeun-Jong Bae, Eunjin Cho, Hunseung Kang

**Affiliations:** ^1^Department of Plant Biotechnology, College of Agriculture and Life Sciences, Chonnam National UniversityGwangju, South Korea; ^2^Department of Bioenergy Science and Technology, College of Agriculture and Life Sciences, Chonnam National UniversityGwangju, South Korea

**Keywords:** *Arabidopsis*, glutathione, heavy metals, Hg accumulation, Hg tolerance, ROS scavenging

## Abstract

Despite the increasing understanding of the crucial roles of glutathione (GSH) in cellular defense against heavy metal stress as well as oxidative stress, little is known about the functional role of exogenous GSH in mercury (Hg) tolerance in plants. Here, we provide compelling evidence that GSH contributes to Hg tolerance in diverse plants. Exogenous GSH did not mitigate the toxicity of cadmium (Cd), copper (Cu), or zinc (Zn), whereas application of exogenous GSH significantly promoted Hg tolerance during seed germination and seedling growth of *Arabidopsis thaliana*, tobacco, and pepper. By contrast, addition of buthionine sulfoximine, an inhibitor of GSH biosynthesis, severely retarded seed germination and seedling growth of the plants in the presence of Hg. The effect of exogenous GSH on Hg specific tolerance was also evident in the presence of other heavy metals, such as Cd, Cu, and Zn, together with Hg. GSH treatment significantly decreased H_2_O_2_ and O_2_^-^ levels and lipid peroxidation, but increased chlorophyll content in the presence of Hg. Importantly, GSH treatment resulted in significantly less accumulation of Hg in *Arabidopsis* plants, and thin layer chromatography and nuclear magnetic resonance analysis revealed that GSH had much stronger binding affinity to Hg than to Cd, Cu, or Zn, suggesting that tight binding of GSH to Hg impedes Hg uptake, leading to low Hg accumulation in plant cells. Collectively, the present findings reveal that GSH is a potent molecule capable of conferring Hg tolerance by inhibiting Hg accumulation in plants.

## Introduction

Heavy metal pollution is an environmental threat that affects ecological systems and living organisms, including plants, animals, and humans. Excess heavy metal accumulation in plants causes physiological disorders and inhibits plant growth and productivity. Since plants are the major source of human food, efforts to develop edible plants with increased tolerance to and reduced accumulation of heavy metals are important for food safety and avoidance of the risk of heavy metal toxicity. To increase heavy metal tolerance and minimize the accumulation or toxicity of heavy metals in plants, it is necessary to understand the mechanisms of uptake, detoxification, and tolerance. To survive and adapt to heavy metal stress, plants have evolved complex defense strategies, such as reduction in cellular free metal content (e.g., metal exclusion, cell wall binding, chelation, and sequestration) and scavenging of reactive oxygen species (ROS) that are generated by heavy metal exposure (Hall, 2002).

Glutathione (GSH) is a tripeptide (γ-glu-cys-gly) that plays a crucial role in cellular defense against heavy metal stress and oxidative stress as well as in plant growth and development. GSH itself is known as a major reservoir of non-protein reduced sulfur for the chelation of heavy metal ions and as a precursor of phytochelatin (PC), which is a typical metal chelator found in plants that facilitates metal sequestration into vacuoles (Cobbett and Goldsbrough, 2002; Yadav, 2010). Many previous studies demonstrated that GSH application partially rescues the inhibition of seedling growth and embryonic lethality of GSH-deficient *Arabidopsis thaliana* mutants (Cairns et al., 2006; Pasternak et al., 2008; Lim et al., 2011). In addition, it has been demonstrated that overexpression of γ-glutamylcysteine synthetase (γ-GCS) and glutathione synthetase (GS), the essential enzymes for GSH biosynthesis, resulted in improved tolerance against various heavy metals in transgenic plants (Zhu et al., 1999a,b; Arisi et al., 2000). Importantly, previous studies showed that heavy metal tolerance is closely related to alterations in heavy metal accumulation. Overexpression of plant or *E. coli* γ-GCS and GS enhances the accumulation of cadmium (Cd) and mercury (Hg) (Zhu et al., 1999a,b; Bittsánszky et al., 2005; Guo et al., 2008; Reisinger et al., 2008), whereas overexpression of γ-GCS or GS in *Brassica juncea* and *A. thaliana* decreases the accumulation of arsenic (As) and Hg (Li et al., 2006).

In addition to these transgenic approaches, many studies have been carried out to elucidate the function of GSH in heavy metal tolerance by directly applying GSH in the presence of heavy metals. Importantly, the effects of exogenous GSH on heavy metal tolerance and accumulation depend on plant species and type of metal. Exogenous GSH enhanced Cd tolerance in oilseed rape (Nakamura et al., 2013), barley (Chen et al., 2010), and rice (Cai et al., 2010; Sun et al., 2013) and led to a decrease in Cd accumulation in the plants. By contrast, exogenous GSH alleviated chromium (Cr) and lead (Pb) stress but caused Cr and Pb accumulation in several plant species, such as rice, maize, barley, and *Iris lactea* (Wang et al., 2011; Zeng et al., 2012; Yuan et al., 2015). In addition, exogenous application of GSH in cultured tobacco, *Rauvolfia serpentina*, and *Sphagnum squarrosum* cells resulted in enhanced tolerance against As, Cd, copper (Cu), and zinc (Zn) and increased the accumulation of Cd and Cu (Nakazawa et al., 2000; Schmöger et al., 2000; Saxena and Saxena, 2012). These findings indicate that exogenous application of GSH confers heavy metal tolerance, and that heavy metal tolerance is not always correlated with the accumulation of heavy metals in plants, which makes further analysis of the functional role of exogenous GSH with respect to metal tolerance and accumulation in plants necessary.

Mercury is one of the most hazardous heavy metals, even at relatively low concentrations, in plant cells (Chen and Yang, 2012), and causes Minamata disease, a neurological syndrome that results from damage to the lungs, kidneys, and muscles of humans following the consumption of Hg-contaminated foods (Vallee and Ulmer, 1972). Mercury is known to induce oxidative stress generated by ROS, which causes lipid peroxidation, enzyme inactivation, and DNA and membrane damage (Cargnelutti et al., 2006; Ortega-Villasante et al., 2007; Zhou et al., 2007), and inhibits photosynthesis, transpiration, and nutrient transport in plants (Patra and Sharma, 2000; Patra et al., 2004). Despite the potent toxicity of Hg during plant growth and development, little is known about the mechanism of Hg tolerance and accumulation in plants. In particular, the effects of exogenous GSH on Hg tolerance in plants are largely unknown. A previous report demonstrated that exogenous GSH positively affects rice seedling growth in the presence of Hg (Mishra and Choudhuri, 1998). To further understand the role of GSH in heavy metal tolerance, we herein assessed the effects of exogenous GSH on seed germination and seedling growth of various plants in the presence of Cd, Cu, Hg, and Zn. We provide compelling evidence that exogenous GSH confers tolerance against Hg stress but not against stress induced by other heavy metals by impeding Hg uptake via the formation of stable GSH-Hg complexes.

## Materials and Methods

### Plant Materials and GSH or BSO Treatment

*Arabidopsis thaliana* (Col-0 ecotype) and tobacco (*Nicotiana tabacum* var. *Xanthi*) seeds were surface-sterilized with 70% ethanol for 1 min and 1% hypochlorite for 5 min, and then rinsed five times with sterilized water. To determine the effects of GSH and buthionine sulfoximine (BSO) on seed germination and seedling growth, seeds were germinated and grown on half-strength MS medium (Murashige and Skoog, 1962) containing 50–200 μM GSH or BSO. The plants were grown at 22°C under long-day conditions (16 h-light/8 h-dark cycle).

### Heavy Metal Tolerance Assay

To evaluate the effects of heavy metals on seed germination, seeds were sown on half-strength MS medium supplemented with 75 μM CdCl_2_, 75 μM CuSO_4_, 750 μM ZnSO_4_, or 10–40 μM HgCl_2_. To test the effects of Hg on seedling growth, the seeds were fully germinated on normal MS medium, 5-day-old seedlings were transferred to MS medium containing various concentrations of HgCl_2_, and root length was measured. To investigate the effects of GSH on multiple types of heavy metal stress, *Arabidopsis* seeds were germinated in the presence of Cd, Cu, Zn, or Hg with or without 50 μM GSH.

### ROS Determination

Approximately 200 mg of 10-day-old seedlings treated with or without GSH in the presence of 10 or 20 μM Hg were extracted with 0.1% trichloroacetic acid (TCA) at 4°C, and the extract was mixed with 0.5 ml of 100 mM potassium phosphate buffer (pH 7.0) and 1 ml of 1 M KI. The reaction mixture was placed in the dark for 1 h, and the H_2_O_2_ content was determined by measuring absorbance at 410 nm. *In situ* accumulation of H_2_O_2_ and O_2_^-^ was detected by histochemical staining with diaminobenzidine (DAB) and NBT, respectively, according to the procedure described by Romero-Puertas et al. (2004) with minor modifications. Briefly, 10-day-old *Arabidopsis* seedlings grown on 1/2 MS agar medium and 3-week-old *Arabidopsis* leaves were carefully removed, transferred to solution containing 20 μM Hg either with or without GSH, and further incubated for 1 day. To detect H_2_O_2_, the seedlings and leaves were immersed in DAB solution (1 mg mL^-1^) and incubated at room temperature for 7 h under continuous light until brown spots developed, which are derived from the reaction of DAB with H_2_O_2_. After de-staining by soaking in 100% ethanol solution, the color of the leaves and roots was observed under a microscope. For O_2_^-^ detection, the leaves were immersed in NBT solution (1 mg mL^-1^) dissolved in 10 mM phosphate buffer (pH 7.8) at room temperature. The immersed leaves were illuminated for 2 h until dark spots appeared, which are characteristic of blue formazan precipitates.

### Measurement of Lipid Peroxidation and Chlorophyll Content

Lipid peroxidation and chlorophyll content were measured in the same samples used for H_2_O_2_ detection as previously described (Kim et al., 2013). Lipid peroxidation was determined by measuring malondialdehyde (MDA) content using the thiobarbituric acid (TBA) method. Briefly, harvested seedlings were homogenized with 0.5% TBA in 20% TCA, and the mixture was incubated at 95°C and then cooled. Absorbance of the supernatant was measured at 532 and 600 nm. To measure chlorophyll content, the seedlings were extracted with 95% (v/v) ethanol, and absorbance of the extract was measured at 664 and 647 nm.

### Determination of Heavy Metal Content

Fourteen-day-old *Arabidopsis* seedlings grown in hydroponic culture were treated with 10 or 20 μM Hg with or without GSH for 1 day and harvested for Hg content analysis. In addition, seeds were sown and grown for 14 days on half-strength MS medium containing 10–20 μM HgCl_2_, 75 μM CdCl_2_, 75 μM CuSO_4_, or 750 μM ZnSO_4_ with or without GSH, and the seedlings were harvested for metal content analysis. Harvested seedlings were thoroughly rinsed with H_2_O, dried at 80°C, and used to measure metal content using an inductively coupled plasma optical emission spectrometer (ICP-OES). All experiments were repeated at least three times.

### Statistical Analysis

Data represent mean values with standard deviations of three independent replications, and statistical significance was analyzed by Student’s *t*-test (*p* < 0.05; SIGMAPLOT software; systat software Inc.).

### Thin Layer Chromatography

Glutathione was reacted with HgCl_2_, CdCl_2_, CuCl_2_, ZnCl_2_, NiCl_2_, or PbCl_2_ (molar ratio, 1:2) for 1 h at room temperature. The mixture was then spotted on a TLC plate with silica gel G. After drying at room temperature, the TLC plate was developed with buthanol/acetic acid/water (3:1:1, v/v/v).

### ^1^H NMR Experiment

Samples containing GSH alone (4 × 10^-3^ M) or GSH together with heavy metals, including Cd^2+^, Cu^2+^, Hg^2+^, and Zn^2+^, were prepared by adding 1 equivalent amount of cations to the GSH solution in D_2_O. ^1^H NMR spectra were recorded on a 300 MHz Varian Unity spectrometer using tetramethylsilane (TMS) as an internal standard.

## Results

### Exogenous GSH Delays Seed Germination and Seedling Growth of *Arabidopsis*

To evaluate the effects of GSH on seed germination and seedling growth, *Arabidopsis* seeds were sown on half-strength MS medium supplemented with various concentrations of GSH, as well as BSO that inhibits a key enzyme necessary for GSH biosynthesis and decreases cellular GSH levels (Griffith and Meister, 1979; Howden et al., 1995a,b). Both GSH and BSO delayed seed germination and inhibited post-germination seedling growth in a concentration-dependent manner (Supplementary Figure S1). When seeds were germinated and grown with a low concentration of 50 μM GSH or BSO, the germination rate and primary root growth of *Arabidopsis* plants were comparable to those of untreated plants, whereas the germination rate and root growth of *Arabidopsis* plants were severely inhibited when treated with 100–200 μM GSH or BSO. Since application of 50 μM GSH or BSO did not affect seed germination and seedling growth of *Arabidopsis*, we used 50 μM GSH or BSO to evaluate the effects of GSH on heavy metal tolerance in subsequent experiments.

### Exogenous GSH Does Not Affect Tolerance to Cd, Co, Cu, Ni, Pb, or Zn

**FIGURE 1:**
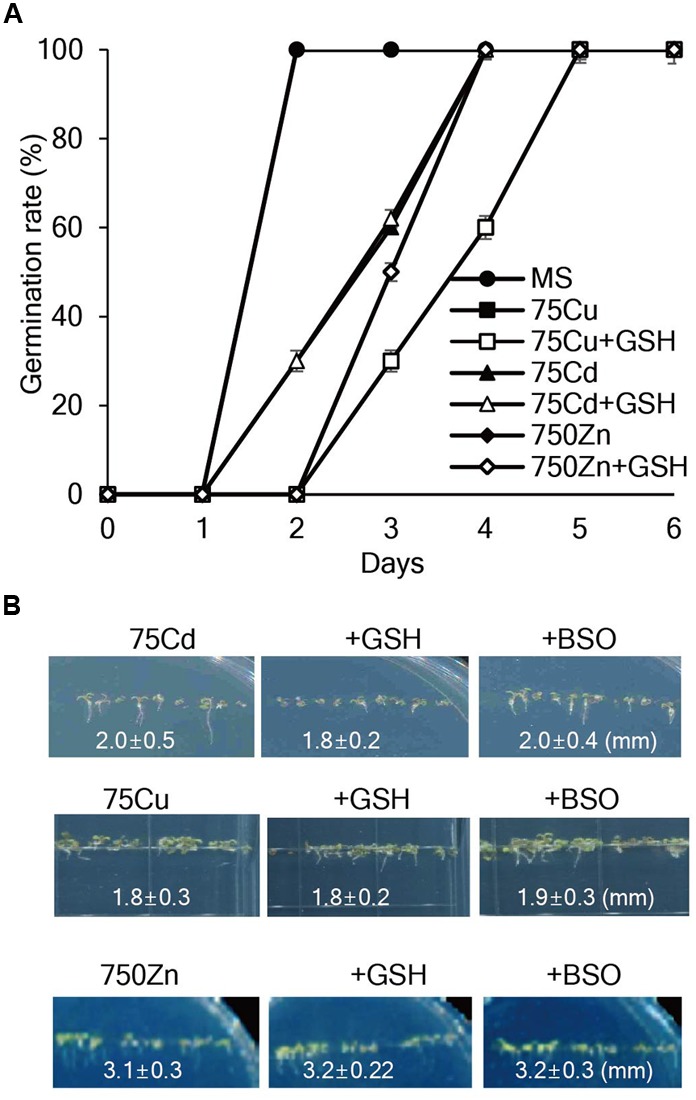
**Effects of GSH on seed germination and seedling growth of *Arabidopsis* in the presence of Cd, Cu, or Zn. (A)** *Arabidopsis* seeds were germinated on MS medium supplemented with 75 μM Cd, 75 μM Cu, or 750 μM Zn with or without 50 μM GSH, and germination rates were scored on the indicated days. **(B)** Post-germination growth of *Arabidopsis* was observed on MS medium supplemented with Cd, Cu, or Zn with or without 50 μM GSH or 50 μM BSO; the photographs were taken on day 7.

To assess whether exogenous GSH contributes to heavy metal tolerance, seed germination, and seedling growth of *Arabidopsis* plants were evaluated on MS medium containing various concentrations of heavy metals with or without GSH. The results showed that 75 μM Cd, 75 μM Cu, or 750 μM Zn markedly inhibited the germination rates and post-germination seedling growth of *Arabidopsis*; root length of 7-day-old *Arabidopsis* was approximately 2.0, 1.8, and 3.1 mm in the presence of 75 μM Cd, 75 μM Cu, or 750 μM Zn, respectively, and application of GSH or BSO did not influence the inhibitory effects of these heavy metals on seed germination and seedling growth (**Figure 1**). Moreover, exogenous GSH did not affect the seedling growth of *Arabidopsis* in the presence of other heavy metals, such as 100 μM Ni, 500 μM Pb, and 500 μM Co (Supplementary Figure S2). These results suggest that exogenous GSH does not affect *Arabidopsis* tolerance against all these tested heavy metals.

### Exogenous GSH Markedly Alleviates Hg Toxicity during Seed Germination and Seedling Growth

**FIGURE 2:**
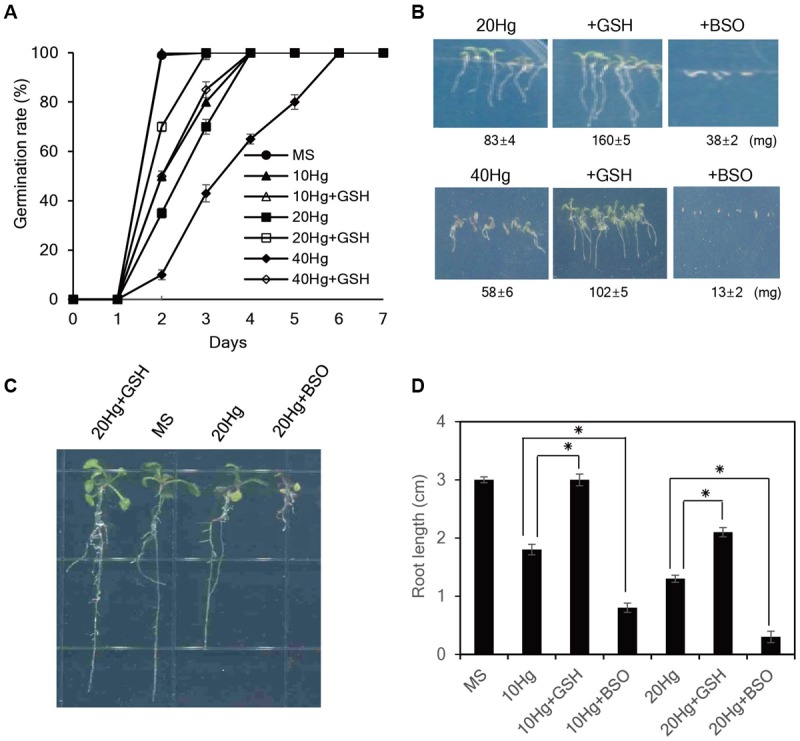
**Effects of GSH on Hg tolerance in *Arabidopsis*. (A)** *Arabidopsis* seeds were germinated on MS medium containing 10–40 μM Hg with or without 50 μM GSH, and germination rates were scored on the indicated days. **(B)** The fresh weight of *Arabidopsis* was measured 7 days after germination on MS medium containing 20 or 40 μM Hg with or without 50 μM GSH or 50 μM BSO. **(C)** Five-day-old seedlings germinated on normal MS medium were transferred to MS medium containing 20 μM Hg with or without 50 μM GSH or 50 μM BSO, grown for 5 days, and **(D)** root length was measured. Data represent the mean ± SD obtained from three biological replicates, and asterisks indicate statistically significant differences between control and treated samples (*t*-test, *p* < 0.05).

We next evaluated whether exogenous GSH confers tolerance to Hg, which is one of the most toxic metals and seriously damages plant growth (Chen and Yang, 2012). As expected, Hg severely retarded seed germination and post-germination seedling growth of *Arabidopsis* plants (**Figure 2**). Notably, exogenous GSH significantly increased Hg tolerance; application of 50 μM GSH markedly promoted seed germination and seedling growth of *Arabidopsis* in the presence of 20–40 μM Hg, while 50 μM GSH treatment completely abolished Hg toxicity at a low (10 μM) concentration (**Figures 2A,B**). By contrast, the seed germination and post-germination seedling growth of *Arabidopsis* plants were more strongly inhibited by the application of 50 μM BSO, an inhibitor of GSH biosynthesis, in the presence of Hg (**Figure 2B**). The positive effects of GSH on seed germination and post-germination growth in the presence of Hg were also observed in other plant species, including tobacco, camelina, rice, alfalfa, and rape seed (Supplementary Figure S3). These results indicate that exogenous GSH confers Hg tolerance in diverse plant species.

To further evaluate the influence of GSH on Hg tolerance, 5-day-old seedlings grown on normal MS medium were transferred to MS medium containing Hg with or without GSH or BSO, and root growth was examined. The results showed that exogenous GSH significantly alleviated Hg-induced toxicity in the presence of 10–20 μM Hg. In particular, GSH treatment completely abolished Hg toxicity at a low (10 μM) concentration; root length of *Arabidopsis* 5 days after 10 μM Hg treatment was approximately 1.8 cm, whereas root length of the plants under 10 μM Hg with GSH was approximately 3.0 cm which was comparable with that grown under normal conditions (**Figures 2C,D**). By contrast, application of BSO further increased Hg toxicity; root length of *Arabidopsis* under 10 μM Hg with 50 μM BSO was approximately 0.8 cm (**Figures 2C,D**). The contribution of GSH to Hg tolerance was also observed in other plant species, such as tobacco and pepper (Supplementary Figure S4). Collectively, these results indicate that exogenous GSH confers Hg tolerance in diverse plant species.

### Exogenous GSH Confers Hg-specific Tolerance in the Presence of Multiple Heavy Metals

**FIGURE 3:**
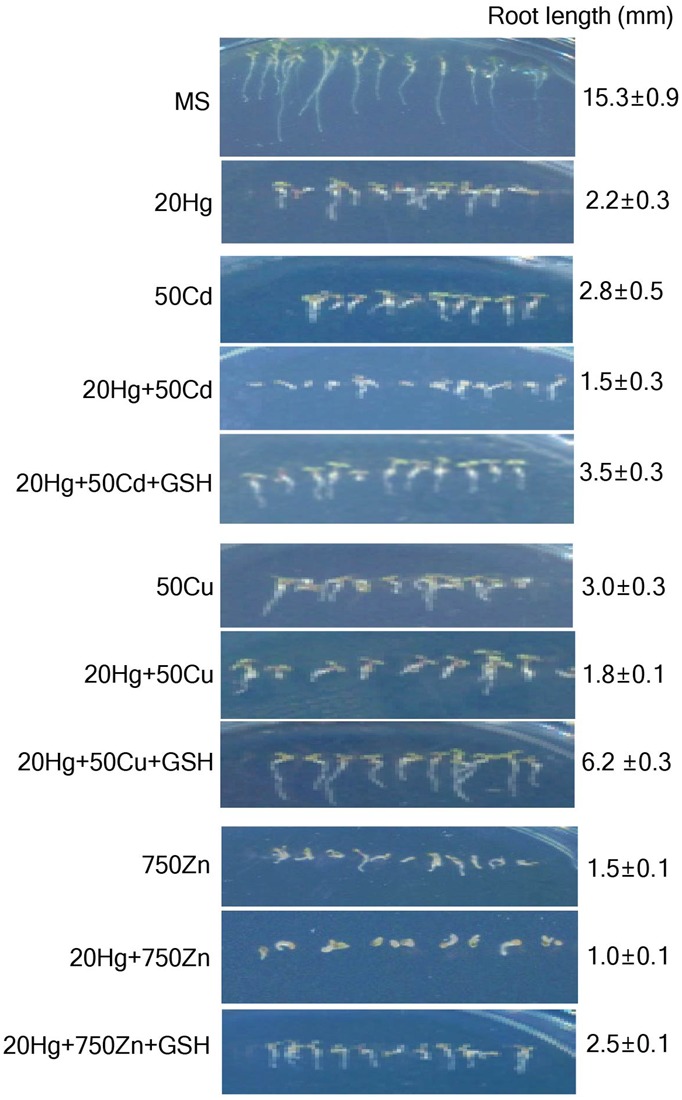
**Effects of GSH on Hg tolerance in the presence of other heavy metals.** *Arabidopsis* seeds were sown and grown in medium containing various combinations of heavy metals and 20 μM Hg with or without 50 μM GSH. Photographs were taken on day 7, and root length of the plants was measured. Data represent the mean ± SD obtained from three biological replicates.

To determine whether GSH confers tolerance specifically to Hg in the presence of other heavy metals, *Arabidopsis* seeds were sown and grown on MS medium supplemented with various combinations of 20 μM Hg and other heavy metals, including 50 μM Cd, 50 μM Cu, or 750 μM Zn, with or without 50 μM GSH. The results showed that *Arabidopsis* root growth was more severely inhibited in the presence of both Hg and each additional heavy metal than in the presence of Hg or each heavy metal alone on day 7 (**Figure 3**). Clearly, GSH treatment significantly increased the root growth of *Arabidopsis* plants in the presence of both Hg and each of the other heavy metals (**Figure 3** and Supplementary Figure S5). These positive effects of GSH on Hg tolerance in the presence of other heavy metals were more clearly visible on day 14 (Supplementary Figure S6). Taken together, these results indicate that GSH contributes to Hg tolerance in the presence of other heavy metals.

### Exogenous GSH Decreases H_2_O_2_ and O_2_^-^ Levels under Hg Stress

**FIGURE 4:**
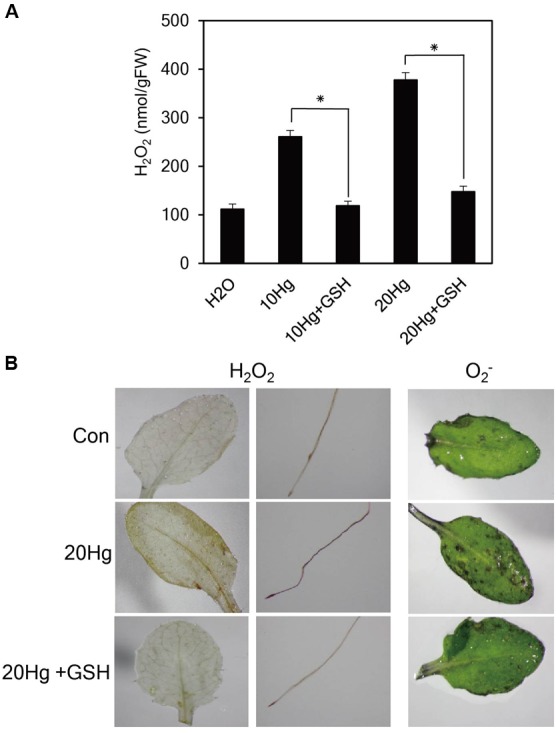
**Effects of GSH on ROS levels. (A)** H_2_O_2_ levels were determined in 10-day-old *Arabidopsis* seedlings treated with 10 or 20 μM Hg with or without 50 μM GSH for 1 day. Data represent the mean ± SD obtained from three biological replicates, and asterisks indicate statistically significant differences between control and treated samples (*t*-test, *p* < 0.05). **(B)** The levels of H_2_O_2_ and O_2_^-^ were detected by histochemical staining using DAB and NBT in 10-day-old *Arabidopsis* seedlings and 3-week-old *Arabidopsis* leaves, respectively, treated with 20 μM Hg with or without 50 μM GSH for 12 h.

Heavy metals are well-known to generate ROS. Since GSH are responsible for reducing the toxic effects of ROS in plant tissues, we examined H_2_O_2_ levels after treatment of Hg with or without GSH. The levels of H_2_O_2_ were markedly increased under 10 or 20 μM Hg stress but were significantly diminished by GSH application (**Figure 4A**). The results were further supported by histochemical detection of H_2_O_2_ (**Figure 4B**). More intense brown formazan precipitates were detected in the leaves and roots of *Arabidopsis* after Hg treatment, whereas the brown signals were relatively weak after GSH treatment (**Figure 4B**). We also detected the superoxide anion (O_2_^-^) in mature leaves of *Arabidopsis*. More intense purple precipitates were observed in mature leaves of *Arabidopsis* after Hg treatment, whereas the purple signals decreased to control levels after GSH treatment (**Figure 4B**). These results indicate that Hg-produced H_2_O_2_ and O_2_^-^ were efficiently scavenged by GSH treatment.

### Exogenous GSH Increases Chlorophyll Content and Decreases Lipid Peroxidation under Hg Stress

**FIGURE 5:**
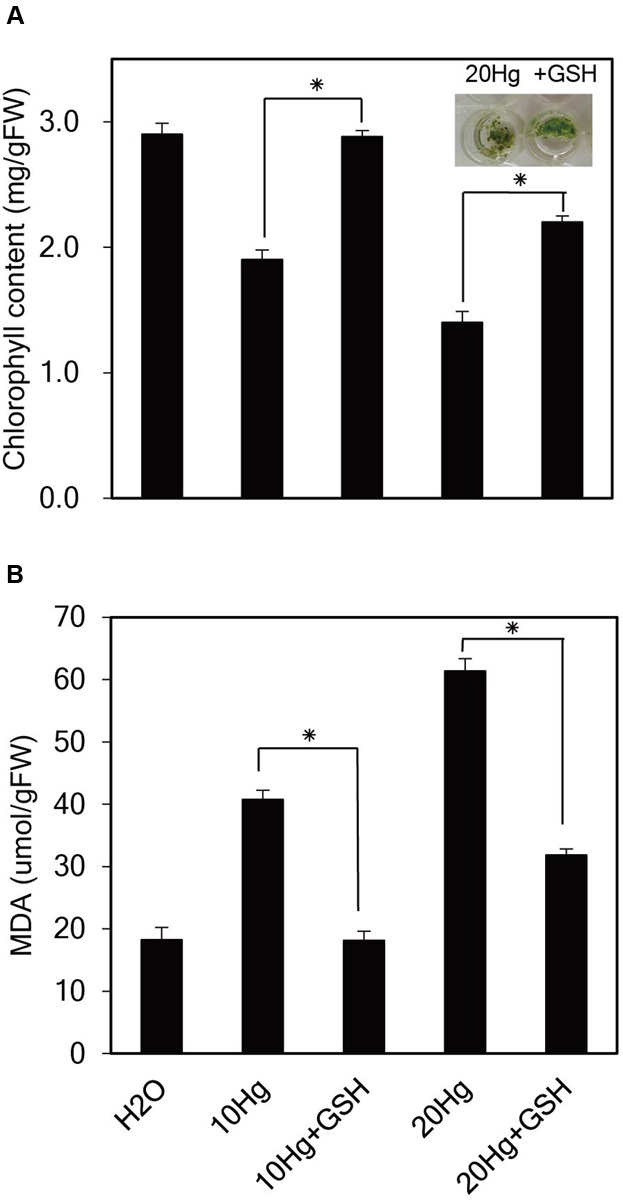
**Effects of GSH on chlorophyll content and lipid peroxidation under Hg stress.** Ten-day-old *Arabidopsis* seedlings were treated with 10 or 20 μM Hg with or without 50 μM GSH for 1 day. Chlorophyll content **(A)** and the levels of malondialdehyde (MDA) **(B)** were determined, and the data show the mean ± SD obtained from three biological replicates. Asterisks indicate statistically significant differences between control and treated samples (*t*-test, *p* < 0.05).

To investigate the physiological effects of exogenous GSH against Hg stress, chlorophyll content, and degree of lipid peroxidation were analyzed in *Arabidopsis* seedlings under Hg stress with or without GSH. When *Arabidopsis* seedlings were incubated in the presence of 10 or 20 μM Hg for 1 day, the color of the leaves changed from green to yellow, whereas the color of the leaves remained green under treatment of Hg plus GSH (**Figure 5A**). The chlorophyll content was decreased to 52 and 31% of the control under 15 and 30 μM Hg exposure, respectively, whereas GSH treatment increased the chlorophyll content up to 99 and 64% of the control in the presence of 10 and 20 μM Hg, respectively (**Figure 5A**). These results indicate that GSH can ameliorate Hg-induced damage on the cellular level. The MDA level is generally used to reflect the extent of membrane lipid peroxidation, which is a sensitive diagnostic index of oxidative damage (Cargnelutti et al., 2006). The levels of MDA were significantly increased in the presence of 10 or 20 μM Hg, whereas MDA levels were markedly decreased by GSH treatment (**Figure 5B**).

### GSH Significantly Decreases Hg Accumulation in *Arabidopsis*

**FIGURE 6:**
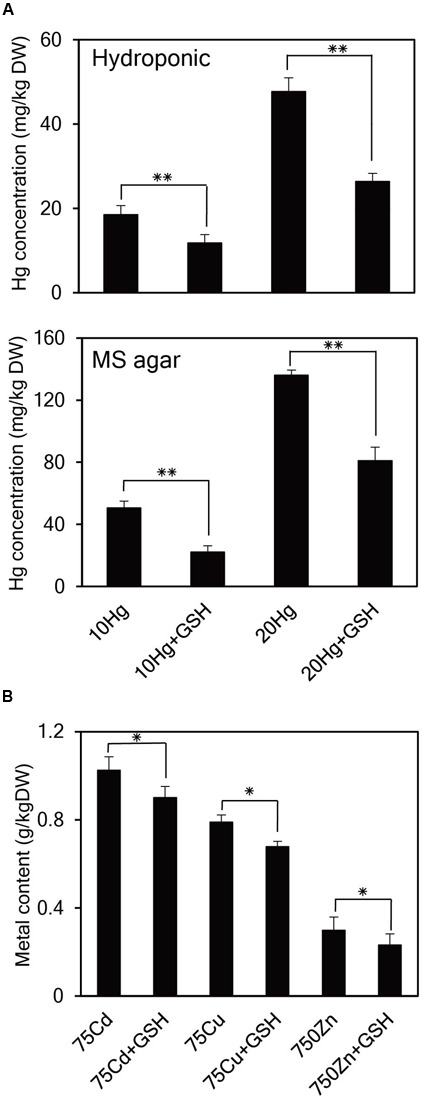
**Effects of GSH on cellular concentrations of Hg and other heavy metals in *Arabidopsis*. (A)** *Arabidopsis* plants grown in hydroponic culture or on MS agar were treated with 10 or 20 μM Hg with or without GSH for 1 day and then harvested for Hg content analysis. **(B)** *Arabidopsis* seeds were germinated on MS medium containing 75 μM Cd, 75 μM Cu, or 750 μM Zn with or without 50 μM GSH. Fourteen-day-old seedlings were used for metal content analysis. Data represent the mean ± SD obtained from three biological replicates, and asterisks indicate statistically significant differences between controls and treated samples (*t*-test, ^∗∗^*p* < 0.01, ^∗^*p* < 0.05).

As it is evident that exogenous GSH alleviates Hg toxicity, the next important question is how GSH confers Hg tolerance in plants. To answer this question, the cellular concentrations of Hg as well as other heavy metals were determined in *Arabidopsis* seedlings grown in the presence of Hg with or without GSH. When 14-day-old *Arabidopsis* plants grown in hydroponic cultures were treated with Hg with or without GSH, Hg content inside the cells reduced down to approximately 64 and 55% levels at 10 or 20 μM Hg, respectively, after GSH treatment (**Figure 6A**). Furthermore, when 14-day-old *Arabidopsis* plants grown on MS agar were treated with Hg with or without GSH, Hg content reduced down to approximately 48 and 60% levels at 10 or 20 μM Hg, respectively, after GSH treatment (**Figure 6A**). On the other hand, exogenous GSH slightly decreased the levels of other heavy metals, such as Cd, Cu, and Zn (**Figure 6B**). These results suggest that GSH much more significantly inhibits the cellular accumulation of Hg than that of other heavy metals in plants.

### GSH Binds Much More Strongly to Hg Than to Other Heavy Metals

**FIGURE 7:**
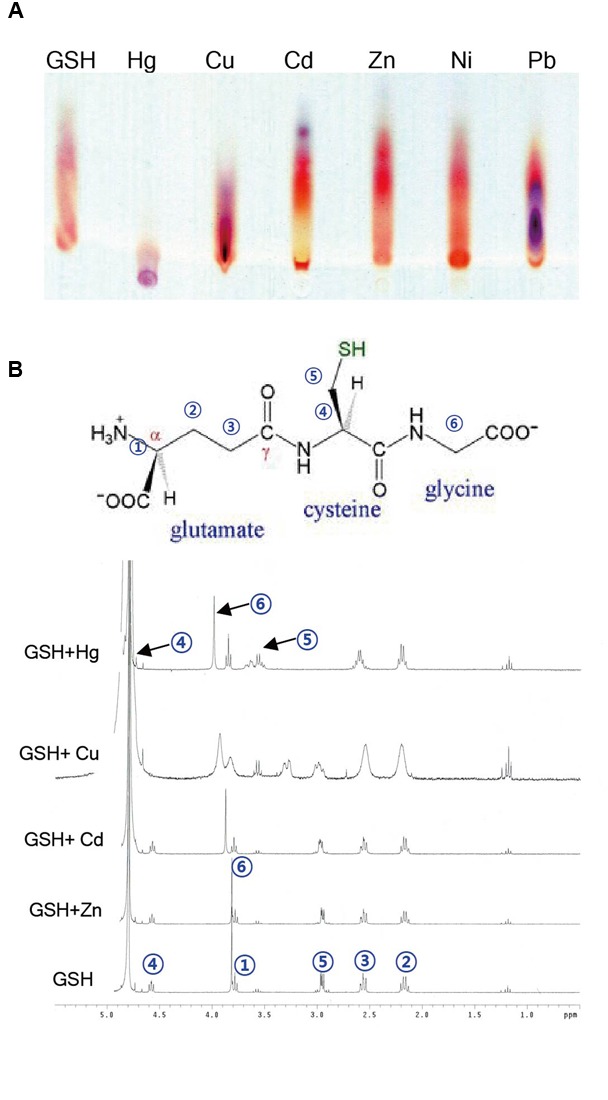
**Analysis of binding between GSH and heavy metals by TLC and NMR. (A)** For TLC analysis, 5 mM GSH was incubated with 5 mM Cd, Cu, Zn, Ni, Pb, or Hg for 1 h. A 2 μl aliquot of each reaction mixture was loaded on a silica gel plate, and after running the plate, the plate was developed with buthanol/acetic acid/water (3:1:1, v/v/v). **(B)** The numbers in a GSH molecule represent each assigned proton. The ^1^H NMR spectra of the mixtures containing 1 equivalent of cations (Cd^2+^, Cu^2+^, and Zn^2+^) along with 4 mM GSH were recorded on a 300 MHz Varian Unity spectrometer using TMS as an internal standard.

Given the evidence that GSH inhibits Hg accumulation in plant cells, the next critical question is how GSH affects the accumulation of heavy metals in plants. To obtain some insight into this important point, we determined the binding affinity of GSH for Hg and other heavy metals using TLC and NMR analysis. First, TLC was used to investigate the migration of GSH-heavy metal complexes on a TLC plate. GSH itself migrated efficiently to the upper part of the plate, whereas Hg significantly suppressed the migration of GSH on the TLC plate (**Figure 7A**). By contrast, the migration of GSH was not affected by other heavy metals, such as Cd, Cu, Zn, Ni, or Pb (**Figure 7A**). The impediment of the migration of GSH in the presence of Hg suggests that GSH forms a stable complex with Hg.

To further determine the binding affinity of GSH for Hg and other heavy metals, we next conducted a ^1^H NMR experiment. The ^1^H NMR spectrum of GSH showed a triplet at δ 4.58 (H4), a singlet at δ 3.82 (H6), a triplet at δ 3.78 (H1), a doublet at δ 2.95 (H5), a triplet at δ 2.56 (H3), and a quartet at δ 2.16 (H2), which can be assigned to each proton in the GSH molecule (**Figure 7B**). We then measured the ^1^H NMR spectra of GSH in the presence of 1 equivalent of HgCl_2_, CdCl_2_, CuCl_2_, or ZnCl_2_. When HgCl_2_ was added to the GSH solution, the chemical shits of three protons, H1, H2, and H3, did not change, whereas a large downfield shift of three protons, H4, H5, and H6, was observed (**Figure 7B**). By contrast, the chemical shifts of the protons in GSH did not change in the presence of other heavy metals, such as Cd, Cu, and Zn, although the addition of Cu caused a small change in the chemical shifts and a broadening of the bands (**Figure 7B**). These results suggest that GSH binds more strongly to Hg than to other heavy metals.

## Discussion

Our findings clearly demonstrate that GSH confers *Arabidopsis* tolerance against Hg but not against other heavy metals, such as Cd, Cu, Zn, Pb, Co, and Ni, which is consistent with previous studies showing that the effects of GSH on heavy metal tolerance depend on plant species and heavy metal type. Our present results show that exogenous GSH has no obvious effects on *Arabidopsis* tolerance to Cd, Cu, Zn, Pb, Co, and Ni (**Figure 1** and Supplementary Figure S1), which is in contrast with previous studies demonstrating that exogenous GSH increases tolerance to these heavy metals in other plant species, including barley, tobacco, and rice (Pomponi et al., 2006; Cai et al., 2010; Wang et al., 2011; Son et al., 2014). Notably, exogenous GSH conferred tolerance specifically against Hg even in the presence of other heavy metals, such as Cd, Cu, and Zn (**Figures 2, 3**). The positive effect of GSH on Hg tolerance was further supported by the observation that application of BSO, an inhibitor of GSH biosynthesis, resulted in a hypersensitive response of *Arabidopsis* to Hg stress (**Figure 2**). Our data clearly show that GSH confers Hg-specific tolerance in diverse plant species, including *Arabidopsis*, tobacco, rice, alfalfa, rapeseed, and pepper.

How does GSH improve Hg tolerance in plants? ROS accumulation is a typical symptom of heavy metal stress, and heavy metal tolerance is closely associated with ROS scavenging. GSH is a key component of the ascorbate (AsA)/GSH cycle, which is associated with H_2_O_2_ scavenging (Noctor and Foyer, 1998; Foyer and Noctor, 2005; Goraya and Asthir, 2016). The contribution of GSH to heavy metal tolerance via ROS scavenging has been demonstrated in various transgenic plants with elevations in endogenous GSH levels (Freeman et al., 2004; Jozefczak et al., 2012 and references therein; He et al., 2015). The present results show that exogenous GSH greatly reduces the levels of H_2_O_2_ and O_2_^-^ that otherwise rapidly accumulate following Hg exposure in *Arabidopsis* roots and leaves (**Figure 4**), implying that exogenous GSH functions as an efficient ROS scavenger. The role of exogenous GSH as an ROS scavenger during Hg stress is also supported by a marked reduction in membrane lipid peroxidation, as evidenced by a low MDA level (**Figure 5B**), which is increased by Hg stress (Cargnelutti et al., 2006; Lomonte et al., 2010; Sahu et al., 2012). It is likely that exogenous GSH rescues cell vitality and plant growth from Hg stress by relieving Hg-induced oxidative stress, which is supported by the observation that *Arabidopsis* leaves maintain their green color and retain a much higher chlorophyll content under Hg stress following the application of exogenous GSH (**Figure 5A**). Collectively, these results suggest that exogenous GSH confers Hg tolerance by relieving heavy metal-induced oxidative stress.

A very important question is how exogenous GSH contributes to tolerance specifically against Hg but not against other heavy metals. It is known that GSH is a key component in the heavy metal scavenging process owing to its high affinity for heavy metals via the interaction with sulfhydryl (-SH) groups. Our current TLC and NMR analyses clearly reveal that GSH has much stronger binding affinity for Hg than for other heavy metals. Suppression of migration of GSH on a TLC plate by Hg but not by other heavy metals, including Cd, Cu, Zn, Ni, or Pb (**Figure 7A**), suggests that GSH forms a stable complex with Hg. Moreover, a large downshift of H4 and H5 protons, which lie in the vicinity of the -SH group, by the addition of Hg but not by other heavy metals (**Figure 7B**) implies that only the Hg ion binds strongly to the -SH group of GSH. Importantly, it has been determined that the stability constant of the Hg-GSH complex is much larger than that of the Cd-GSH complex (Oram et al., 1996; Leverrier et al., 2007; Cardiano et al., 2011), and that thiol-containing peptides form more stable complexes with Hg than with Cd or Pb (Ding et al., 2015). These previous studies and our current results clearly demonstrate that GSH forms much more stable complexes with Hg than with other heavy metals. Based on these observations, we speculate that exogenous GSH binds to Hg and forms stable complexes with Hg outside of plant cells, which results in decreased transport of Hg into plant cells and less accumulation of Hg in *Arabidopsis* (**Figure 6**). Altogether, these results suggest that exogenous GSH confers Hg tolerance by inhibiting Hg entry into plant cells.

In summary, the present study demonstrates that exogenous GSH alleviates Hg toxicity during seed germination and seedling growth of *Arabidopsis* in the presence of concentrations of Hg that far exceed those found in the natural environment. Considering that all the metal ions tested are divalent cations, it would be interesting to further determine the mechanism by which GHS binds more strongly to Hg than to other heavy metals. Because GSH is an efficient chelator of Hg ions, exogenous application of GSH could provide a valuable and economical means to cultivate crop plants in Hg-polluted areas.

## Conclusion

Our results demonstrate that exogenous GSH promotes specifically Hg tolerance during seed germination and seedling growth by decreasing ROS generation and lipid peroxidation. Importantly, GSH has much stronger binding affinity to Hg than to Cd, Cu, or Zn, suggesting that tight binding of GSH to Hg impedes Hg uptake, leading to low Hg accumulation in plant cells. Further studies are needed to determine whether the Hg-GSH complex indeed forms and how entry of the Hg-GSH complex into plant cells is inhibited. Considering that GSH is a potent molecule capable of conferring Hg tolerance by inhibiting Hg accumulation in plants, it would be valuable to further evaluate whether exogenous application of GSH can be a practical means to induce Hg tolerance of diverse plants in heavy metal polluted area.

## Author Contributions

Y-OK: substantial contributions to the conception or design of the work and writing of manuscript. H-JB: drafting the work. EC: experimental analysis. HK: revising for important intellectual content and interpretation of the work.

## Conflict of Interest Statement

The authors declare that the research was conducted in the absence of any commercial or financial relationships that could be construed as a potential conflict of interest. The reviewer PT and handling Editor declared their shared affiliation, and the handling Editor states that the process nevertheless met the standards of a fair and objective review.
