# Supplementary material for: Exogenous Glutathione Enhances Mercury Tolerance by Inhibiting Mercury Entry into Plant Cells
Source: Front Plant Sci. 2017 May 1;8:683. doi: 10.3389/fpls.2017.00683 (PMC5410599; doi:10.3389/fpls.2017.00683)
Supplement: Supplementary file 1 [file Image_1.PDF]

Supplementary Material

Exogenous glutathione enhances mercury tolerance by inhibiting mercury entry into plant cells

Yeon-Ok Kim*, Hyeun-Jong Bae, Eunjin Cho, Hunseung Kang*

***Correspondence:** Hunseung Kang: hskang@jnu.ac.kr & euniceyeonok@hanmail.net

1. Supplementary Data

1.1. Supplementary Figures legends

Supplementary Figure S1. Effects of GSH and BSO on seed germination and seedling growth of *Arabidopsis.* **(A)** *Arabidopsis* seeds were germinated on MS medium supplemented with 50-200 µM GSH or BSO, and germination rates were scored at the indicated days. **(B, C)** Post-germination growth of *Arabidopsis* was observed on MS medium supplemented with 50-200 µM GSH or BSO, and root length was measured on day 7.

Supplementary Figure S2. Effects of GSH on heavy metal tolerance. *Arabidopsis* was grown on MS medium supplemented with 100 µM Ni, 500 µM Pb, or 500 µM Co with or without 50 µM GSH, and root length was measured on day 7.

Supplementary Figure S3. Effects of GSH on heavy metal tolerance in other plant species. The growth of tobacco, camelina, rice, alfalfa, and rapeseed was observed in the presence of 20 µM Hg with or without 50 µM GSH. Photographs were taken on day 7.

Supplementary Figure S4. Effects of GSH on seedling growth of tobacco and pepper under Hg stress. The seeds of tobacco and pepper were germinated on normal MS medium, and 5-day-old tobacco and 3-day-old pepper seedlings were transferred to MS medium containing 10-40 µM Hg with or without 50 µM GSH and BSO. Root length was measured, and photographs were taken on day 5.

Supplementary Figure S5. Effects of GSH on Hg tolerance in the presence of other heavy metals. *Arabidopsis* seeds were sown in the medium containing various combinations of heavy metals and 20 µM Hg with or without 50 µM GSH. Photographs were taken on day 11, and root length of the plants was measured.

Supplementary Figure S6. Effects of GSH on *Arabidopsis* seedling growth in the presence of Hg and other heavy metals. *Arabidopsis* seeds were sown and grown in the medium containing various combinations of heavy metals and 20 µM Hg with or without 50 µM GSH. Root length was measured, and photographs were taken on day 14.

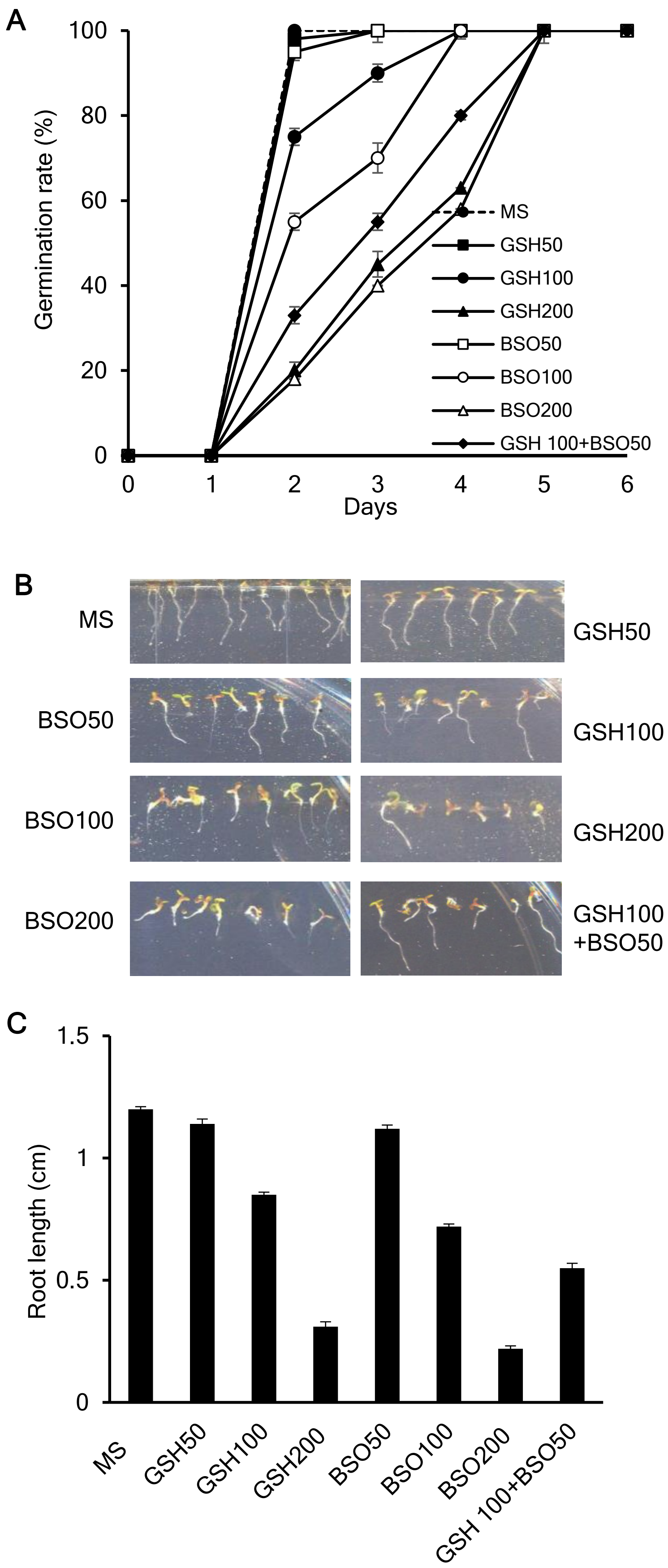

Supplementary Figure S1

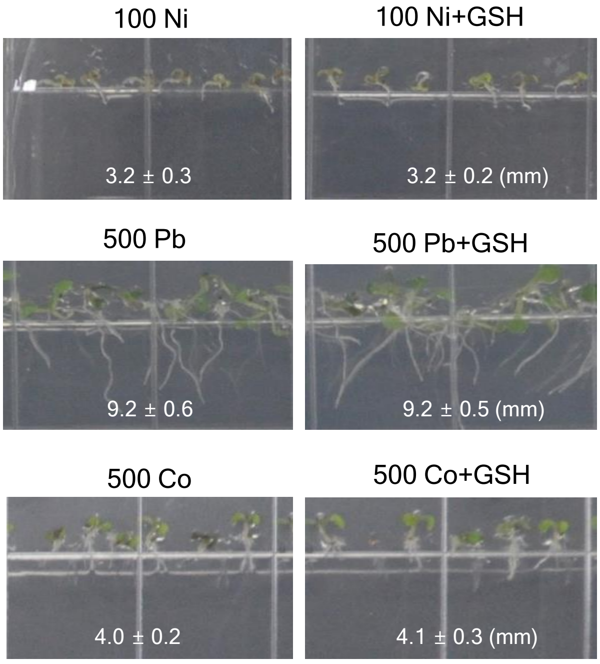

Supplementary Figure S2

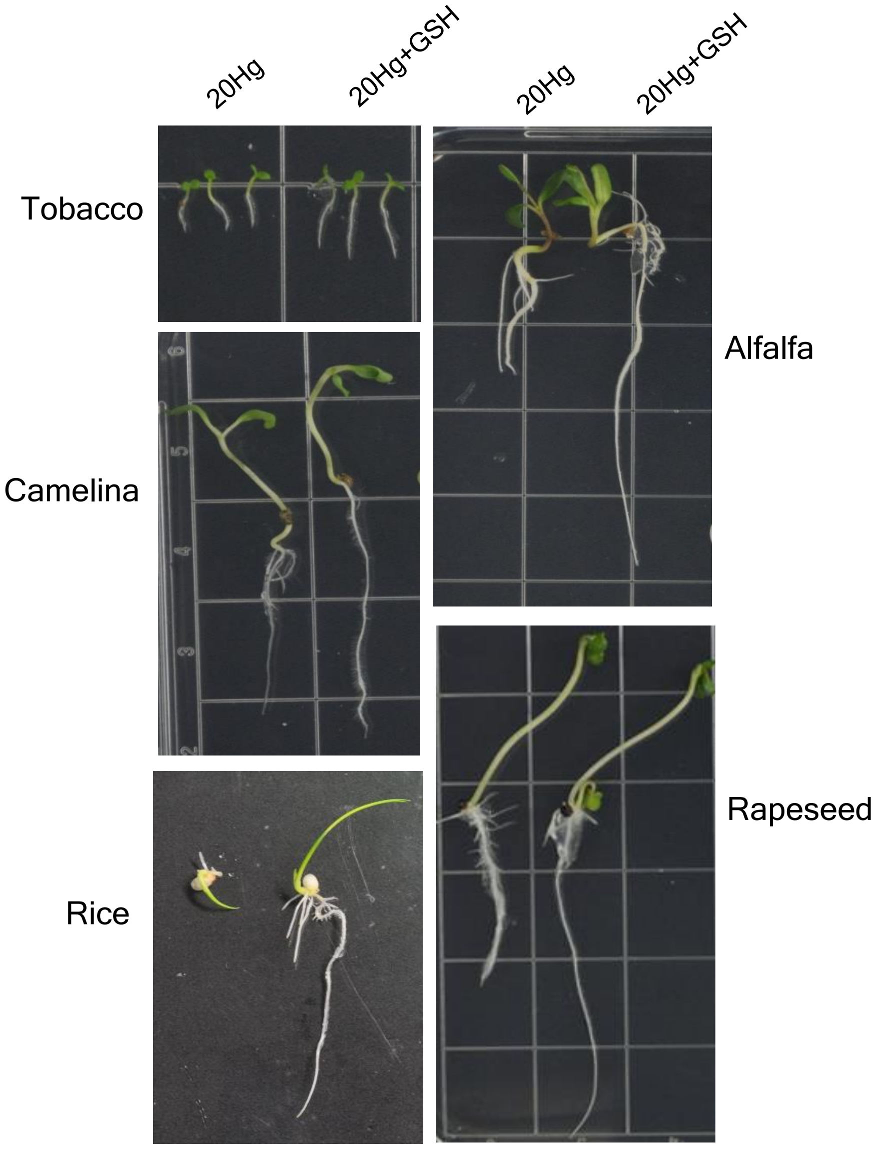

Supplementary Figure S3

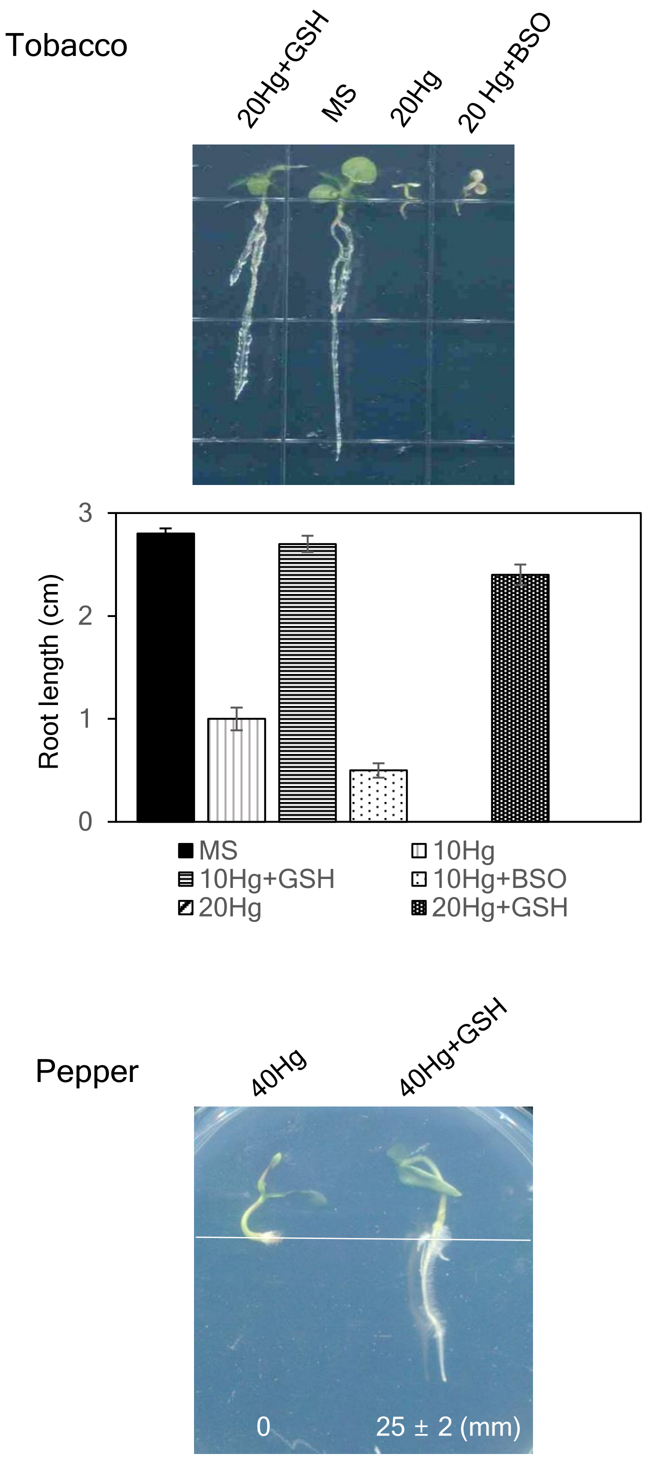

Supplementary Figure S4

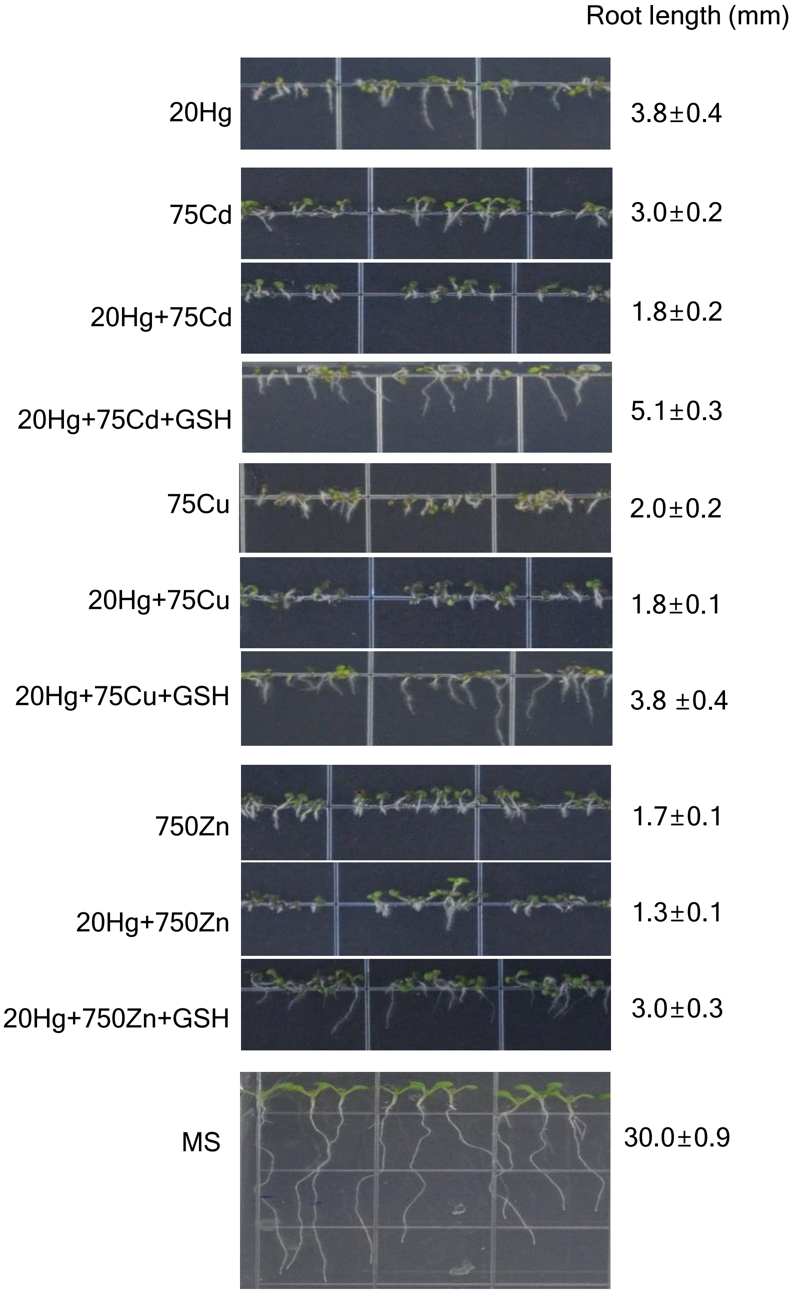

Supplementary Figure S5

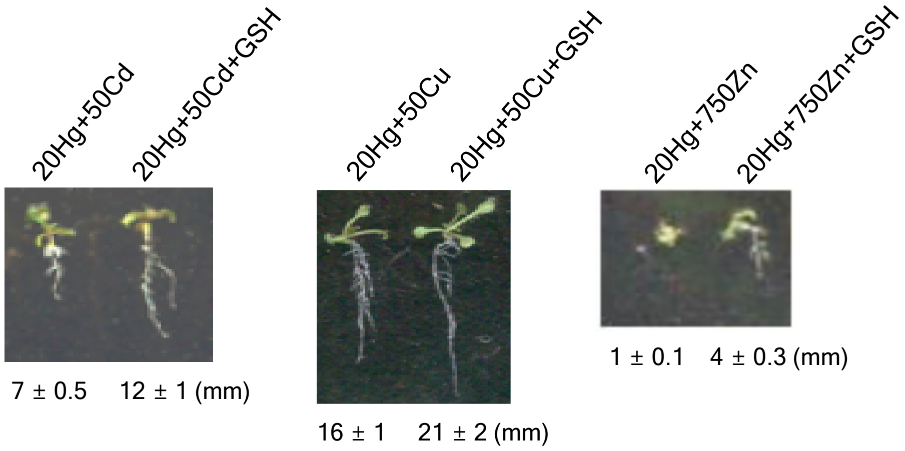

Supplementary Figure S6
